# Brain Complex Network Characteristic Analysis of Fatigue during Simulated Driving Based on Electroencephalogram Signals

**DOI:** 10.3390/e21040353

**Published:** 2019-04-01

**Authors:** Chunxiao Han, Xiaozhou Sun, Yaru Yang, Yanqiu Che, Yingmei Qin

**Affiliations:** Tianjin Key Laboratory of Information Sensing & Intelligent Control, Tianjin University of Technology and Education, Tianjin 300222, China

**Keywords:** driving fatigue, EEG, complex network, functional connectivity, shortest path length, clustering coefficient, degree centrality

## Abstract

Fatigued driving is one of the major causes of traffic accidents. Frequent repetition of driving behavior for a long time may lead to driver fatigue, which is closely related to the central nervous system. In the present work, we designed a fatigue driving simulation experiment and collected the electroencephalogram (EEG) signals. Complex network theory was introduced to study the evolution of brain dynamics under different rhythms of EEG signals during several periods of the simulated driving. The results show that as the fatigue degree deepened, the functional connectivity and the clustering coefficients increased while the average shortest path length decreased for the delta rhythm. In addition, there was a significant increase of the degree centrality in partial channels on the right side of the brain for the delta rhythm. Therefore, it can be concluded that driving fatigue can cause brain complex network characteristics to change significantly for certain brain regions and certain rhythms. This exploration may provide a theoretical basis for further finding objective and effective indicators to evaluate the degree of driving fatigue and to help avoid fatigue driving.

## 1. Introduction

Fatigued driving is a frequent cause of fatal road accidents and is of great concern of the public [1,2,3]. Fatigue is a complex physiological and psychological phenomenon, and driving fatigue is the technical fatigue involved in both mental and physical exertion [4]. It is proved that mental fatigue is a gradual process, and its effects will accumulate as time goes on [5]. Continuous repetitive driving movements for a long time can lead to some physiological and psychological changes for drivers, and then affect their driving ability and alertness [6]. It is considered that fatigue is dominated by the central nervous system, characterized by the body’s reaction in the physiological and chemical metabolism process. Along with the changes in the internal environment of the body and related dysfunction, fatigue gradually affects other body tissues and even the whole body. Many researchers show that mental fatigue induces low vigilance, slow response and drowsiness, which have strong negative impacts on cognitive function [7]. Therefore, it is of great significance to study the evolution process of mental fatigue during driving, which can help to find objective and effective evaluation indicators for fatigue driving and provide a theoretical basis for studying prevention measures.

With the development of brain functional imaging technology, such as electroencephalogram (EEG), functional magnetic resonance imaging (fMRI) and functional near-infrared spectroscopy (fNIRS), the brain dynamics can be recorded. EEG can directly measure the neural electrical activity from different places of the scalp with high time resolution, which is a kind of electrophysiological signal. EEG signals can be divided into delta, theta, alpha and beta rhythms based on the frequency range, which are related to the cognitive functions. EEG signals have obvious rhythmic changes with different degrees of mental fatigue. For driving fatigue, traditional analysis methods such as energy and complexity are most commonly used based on EEG rhythms. It is found that energy based on the power spectrum increases mostly for delta and theta rhythms [8], while the complexity show a downward trend for all rhythms [9]. However, these methods only describe the EEG signals of a single channel in a local brain region, and do not involve the functional connection between brain regions, resulting in great limitations. Therefore, the complex network theory [10,11,12,13,14,15,16,17,18,19] has been introduced into the study of driving fatigue to reveal the functional connectivity of different brain regions and the functional state transition of the whole brain.

Complex network originates from graph theory and consists of the coordination among various elements in the system, which has been widely used in the urban transportation, financial economy, biology, chemistry, power systems and other aspects [20,21,22,23,24]. The nervous system of the brain is a complex network of neurons, which is the basis of various information processing and cognitive expression of the brain. Different brain regions have their own specific functions, but they cannot accomplish tasks alone without cooperating between different brain regions, thereby forming complex networks of interactions. Therefore, driving fatigue may be a complex process involving multiple brain regions. In view of the brain cooperation mechanism, functional connectivity was introduced, which refers to any type of correlation between time series of brain activity [25,26,27]. To some extent, functional connectivity can reflect functional interactions between different brain regions [28]. Accordingly, in our study, the complex network based on functional connectivity from EEG signals is called the “brain functional network”.

At present, there are many studies on driving fatigue based on the complex network [10,11,12,13,14,15,16,17,18,19], but there is no consistent conclusion yet, which will be elaborated on in Discussion section. Therefore, this paper used the complex network theory to further study the changes of brain dynamics from EEG signals in different driving periods during simulated driving at different rhythms. We attempt to provide reliable indicators and a theoretical basis for monitoring the practical application of driving fatigue.

## 2. Materials and Methods 

### 2.1. Subjects

The study included 16 subjects (12 males, 4 females; aged 23 ± 2.9 years), all of whom were college students and graduate students. By the inquiry of experimenters, the subjects must be right-handed and have no history of brain disease (such as pain, schizophrenia, concussion, brain trauma, etc.), ocular disease, psychotropic medication and drug abuse. In addition, the subjects were asked not to drink alcohol, tea, coffee or any other drinks, food or drugs that might excite the central nervous system within 48 hours before the experiment.

### 2.2. Experimental Design

**Figure 1:**
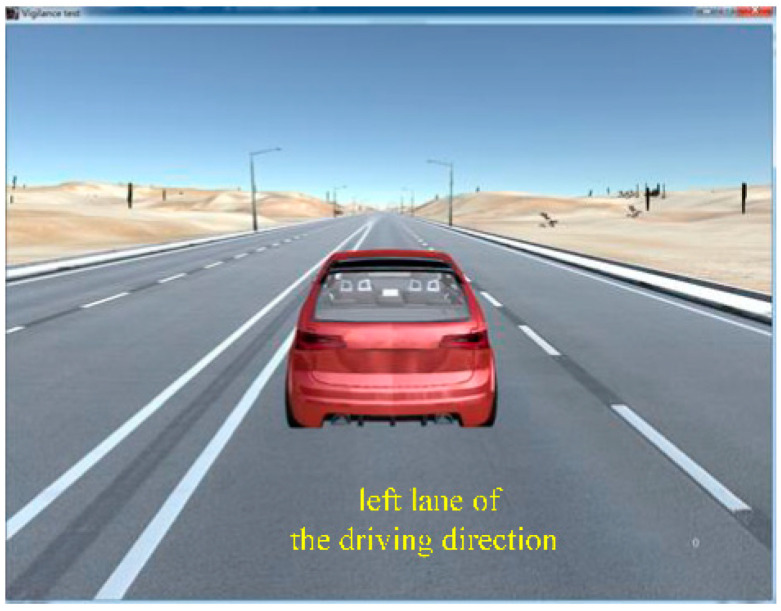
Simulated driving experiment interface.

A simulated driving system was built in this experiment using the game development engine of Unity3D, as shown in Figure 1. The system created a boring driving environment for the subjects to make them more prone to fatigue by using the desert as the background environment. The system was also equipped with the Logitech G29 steering wheel, which used dual-motor force feedback technology to simulate the feedback effect. The entire system strived to maximize the simulation of the real driving environment. This study designed a task of correcting auto-offset of vehicle. The subject should pay attention during the simulated driving process. When the vehicle was automatically offset, the subject needed to adjust the steering wheel in time to keep the vehicle in the middle of the left lane of the driving direction.

**Figure 2:**
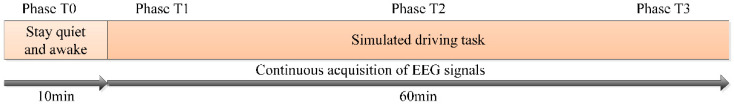
The illustration of the simulated driving experiment procedure.

The environment was comfortable during the experiment, and the light was soft and free of external noise. The total duration of the experiment was 70 min, including the first 10 min to maintain a quiet awake state (phase T0), and the last 60 min of simulated driving state (chose 10 min at the beginning, the middle and the end of simulated driving state as phase T1, T2 and T3, respectively). The detailed process of the experiment is shown in Figure 2. Before the experiment, the subject was informed of the process and requirements of the experiment, clarified that the experiment caused no harm to the body, and then signed the informed consent. The whole experiment process did not violate morality and ethics, and the subjects had a good cooperation attitude. The experiment was carried out following the rules of the Declaration of Helsinki.

### 2.3. EEG Collection and Preprocessing

**Figure 3:**
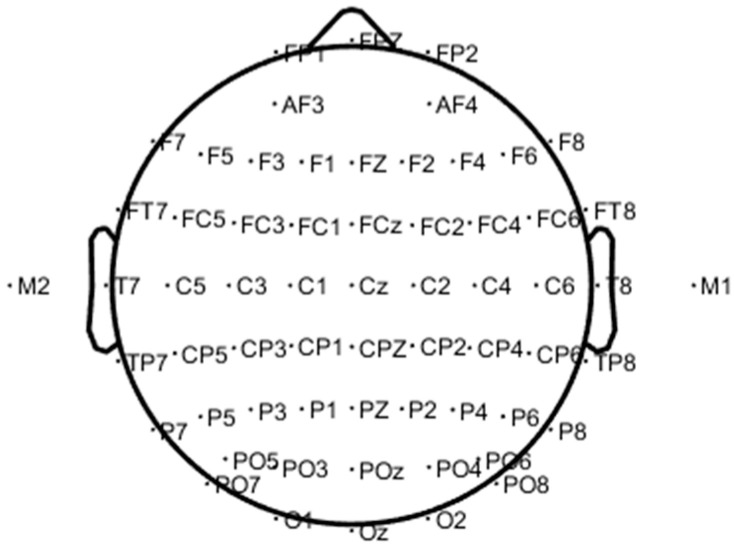
The 62 channel electrode positions of the brain.

The EEG signals of 62 channels were collected in the experiment using the Neuroscan acquisition device, and the electrode position was placed in an international standard 10–20 system as shown in Figure 3. The sampling rate was 1 kHz. EEG channels were distributed in different brain regions, as shown in Table 1. Since the collected EEG signals usually contained interference signals (such as artifacts), the accuracy of the data analysis results would be greatly affected. Therefore, the acquired EEG signals needed to be pre-processed before further analysis. The main preprocessing steps were as follows.

Step 1: Re-reference. The 62-channel EEG data were re-referenced in the experiment. Bilateral mastoids (M1, M2) were used as reference electrodes, so that there were 60 channels in the subsequent analysis.

Step 2: Filtering. After re-reference, a high-pass filter of 0.5 Hz was performed.

Step 3: Down-sampling rate. A 256 Hz sampling reduction process was carried out.

Step 4: Artifact removal. Eye blink, eye drift and head movement artifacts were removed by independent component analysis [29].

Step 5: Dividing frequency band. The EEG rhythms selected in the study were the delta rhythm (0.5–4 Hz), the theta rhythm (4–8 Hz), the alpha rhythm (8–13 Hz), the beta rhythm (13–30 Hz), which were obtained by Equiripple high-pass and low-pass filters.

## 3. Analysis Method

### 3.1. Complex Network Construction

In a complex network, there are two basic elements: nodes and connecting edges. Whether there is a connecting edge between any two nodes depends on the correlation between them. There are a lot of ways to quantify the correlation, such as Pearson correlation, partial correlation, phase synchronization, mutual coherence, and synchronous likelihood method [30,31]. To build a brain functional network based on EEG signals, each channel can be considered as a node, and the functional connectivity is regarded as the connecting edge. Here, we used a standardized sample Pearson correlation coefficient to quantify the functional connectivity between any pair of EEG signals. The calculation formula is as follows:

(1) $$r=\frac{1}{n-1}\sum_{i=1}^{n}\left(\frac{X_{i}-\bar{X}}{s_{X}}\right)\left(\frac{X_{i}-\bar{Y}}{s_{Y}}\right)$$

where $s_{X}$ and $s_{Y}$ are the standard deviations of the samples, respectively, then $\left(\frac{X_{i}-\bar{X}}{s_{X}}\right)$ and $\left(\frac{X_{i}-\bar{Y}}{s_{Y}}\right)$ are the normalized variables, respectively.

In the process of the complex network construction, the adjacency matrix is established for a given threshold T. If r>T, there is a connecting edge between the two nodes, then the corresponding element in the matrix is set to 1; otherwise, there is no connecting edge between the two nodes, and the corresponding element in the matrix is set to 0. Therefore, the adjacency matrix is a binary matrix, and its corresponding network is uniquely determined.

### 3.2. Shortest Path Length

In a complex network, the path length is defined as the number of connecting edges on the path between two nodes $v_{i}$ and $v_{j}$. Although there is usually more than one path between two nodes, only the shortest path is considered because it is the best path for information transmission between nodes. Therefore, the corresponding path length is called the shortest path length [32]. In the brain functional networks, the path length is used to assess the degree of functional integration of the brain regions. The shorter the path length, the stronger the functional integration, that is, the more direct connections between brain regions.

In a binary network, the average shortest path length is defined as:

(2) $$L=\frac{1}{C_{N}^{2}}\underset{1\leq i<j\leq N}{\sum }d_{ij}$$

where $d_{ij}$ represents the shortest path length between node $v_{i}$ and $v_{j}$, and N is the number of nodes in the network.

### 3.3. Clustering Coefficient

In a complex network, the clustering coefficient is an important parameter for measuring the degree of internal grouping and connection, which reflects the possibility of all neighboring nodes of a node being neighbors to each other. The clustering coefficient describes the speed of information processing and transmission from the perspective of the network. 

In a binary network, the clustering coefficient $C_{i}$ of node $v_{i}$ is defined as the ratio of the actual number of sides $E_{i}$ and the total number of possible sides $C_{k_{i}}^{2}$ between $v_{i}$’s adjacent nodes [32], as follows:

(3) $$C_{i}=\frac{E_{i}}{C_{k_{i}}^{2}}$$

where $k_{i}$ is the number of $v_{i}$’s all neighbor nodes, and $E_{i}$ is the actual number of connecting edges between the neighbor nodes of $v_{i}$. The average clustering coefficients of all nodes in the network are defined as follows:

(4) $$C=\frac{1}{N}\overset{N}{\underset{i=1}{\sum }}C_{i}$$

where N is the total number of nodes.

### 3.4. Degree Centrality

In a complex network, the degree centrality is the most direct index to measure the degree of node centrality in the network. For a brain functional network, degree centrality reflects the cerebral cortex regions that play a key role in the information transmission and processing of the brain [33]. The degree centrality of a node is large, which means that the node has more connected nodes in the topology of the brain functional network, indicating that it has an important position in the network. Therefore, the degree centrality can quantitatively analyze the importance of the node in the brain functional network. The degree centrality can be defined as follows:

(5) $$C_{D}=\frac{k_{i}}{N-1}$$

where $k_{i}$ is the degree of node $v_{i}$, that is, the number of edges connected to the node $v_{i}$; N is the total number of nodes.

### 3.5. Statistical Method

In our study, what we were most concerned about was the difference between phase T1, T2 or T3 compared to phase T0. In statistics, phase T0 was the baseline, i.e., the control group, while phase T1, T2 and T3 were the test groups. One-way analysis of variance (ANOVA) was used to compare the complex network index (the amount of functional connectivity, shortest path length, clustering coefficient or degree centrality) extracted from EEG signals between phase T1, T2 or T3 and phase T0. Then, Dunnett’s test followed to correct for multiple comparison under the significant level p<0.05.

### 3.6. Analysis Example

**Figure 4:**
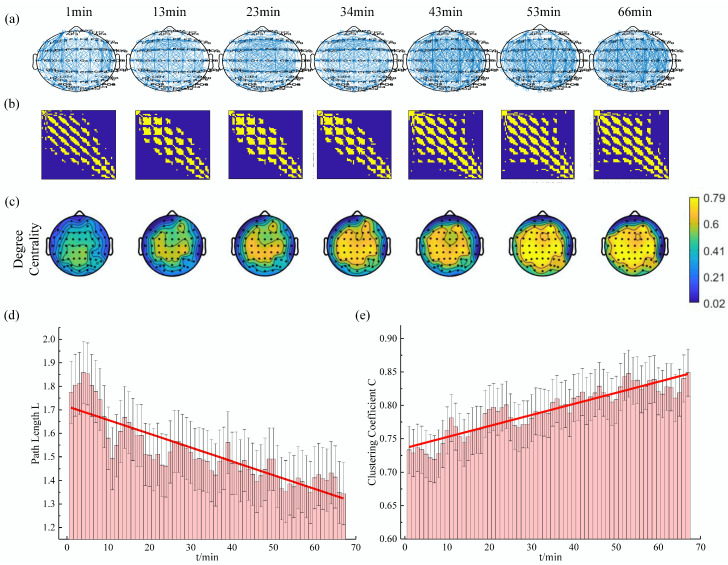
The evolution of the complex network characteristics in the delta rhythm for one subject (T=0.74). (**a**) The functional connectivity graph at several specific times; (**b**) The heatmap of binary brain network at several specific times (The yellow point represents the element with the adjacency matrix of 1.); (**c**) The topographic map of the degree centrality at several specific times; (**d**) and (**e**) are the changes of the average shortest path length and clustering coefficient, respectively. (The red lines are the results of first-order linear quasi-sum based on the mean point of each window.)

In this study, the above analysis methods were applied to calculate the variation of the characteristic parameters in each subject with sliding window technique. The results of one subject at a threshold of 0.74 in the delta rhythm are shown in Figure 4. It can be seen that the changes in the structure of the brain functional network were obvious. At the beginning of the experiment, the brain network of the subjects was sparse, and the elements with the adjacency matrix of 1 were relatively few. With the increase of simulated driving time, the brain network of the subject gradually became denser, and the elements with the adjacency matrix of 1 were relatively more abundant. Meanwhile, the average shortest path length decreased, the average clustering coefficient increased, and the degree centrality of almost the whole brain region increased gradually.

## 4. Results

### 4.1. Threshold Selection

EEG channels were used as nodes of the complex network, and the corresponding node connection was established according to the Pearson correlation coefficient between the two channels. Thereby, the brain functional network based on EEG signals for driving fatigue was constructed.

The selection of the threshold T is directly related to the structure of the complex brain network. Different thresholds may lead to different complex networks, so we traversed all possible thresholds from 0 to 1 with a step of 0.01. 

**Figure 5:**
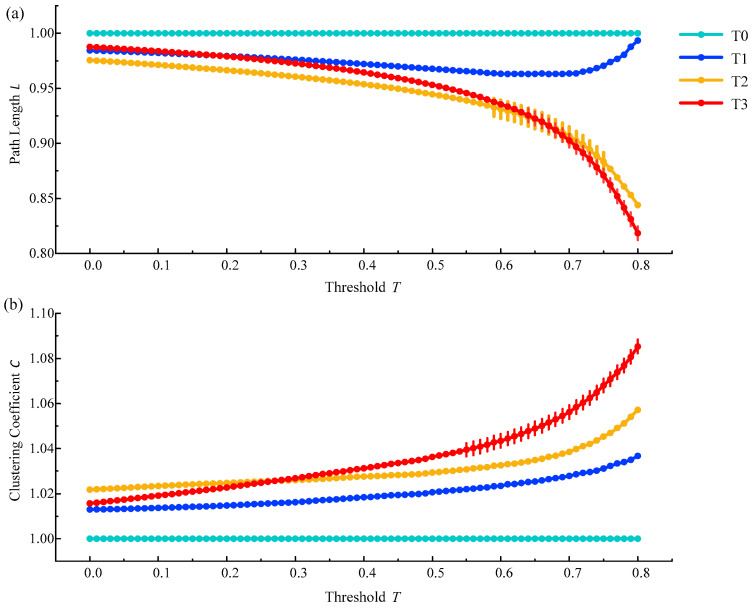
The relative changes of the average shortest path length and the average clustering coefficient in phases T1, T2 and T3 compared to phase T0 in the brain functional network from all subjects in the delta rhythm. (**a**) The relative change of the average shortest path length; (**b**) The relative change of the average clustering coefficient. Vertical lines represent thresholds with significant differences compared to phase T0 (p<0.05).

On the premise of ensuring that there were no isolated nodes or isolated parts in the constructed brain functional network, threshold selection mainly depended on the statistical results of path length and clustering coefficients between phase T1, T2, or T3 compared to phase T0. We tended to choose the threshold interval with significant differences. The statistical analysis method we used here was one-way ANOVA followed by Dunnett’s test (*p* < 0.05). It was found that there were significant difference at the threshold of 0.65–0.77 between phase T2 (and T3) and phase T0 for the shortest path length in the delta rhythm, and at the threshold of 0.56–0.80 between phase T3 and phase T0 for the clustering coefficient in the delta rhythm, as is shown in Figure 5. It was concluded that when the threshold T was in the range from 0.65 to 0.77, the difference between phases T1–T3 and the phase T0 was more obvious. From this, the threshold from 0.65 to 0.77 was finally selected to construct the brain functional network.

### 4.2. Functional Connectivity Analysis

**Figure 6:**
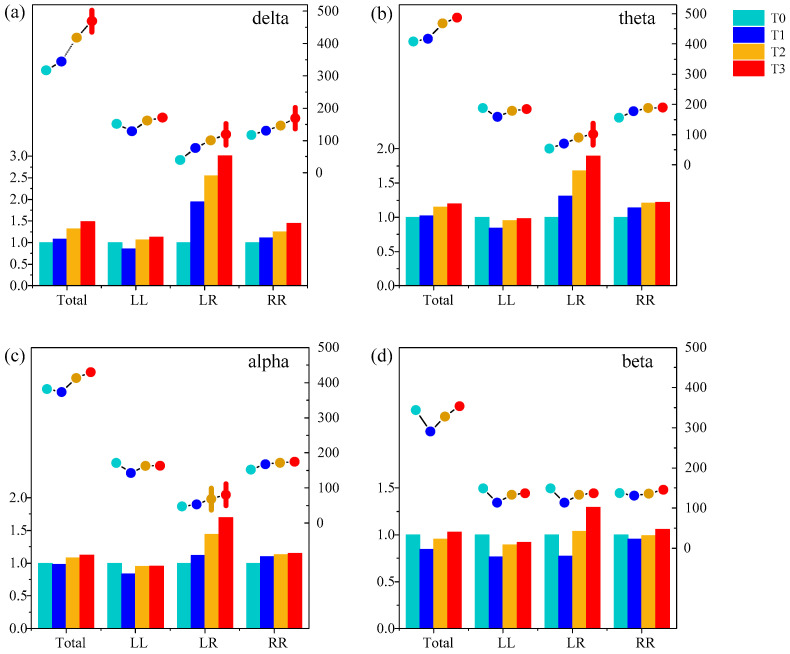
The amount of functional connectivity (line) and the relative change (histogram) of phases T1–T3 compared to phase T0 from all subjects in (**a**) delta, (**b**) theta, (**c**) alpha and (**d**) beta rhythms. Four different colored shaded columns represent different phases. The histogram corresponds to the bottom and left axis, while the line corresponds to the bottom and right axis. Vertical lines represent the amount of functional connectivity with significant differences compared to phase T0 (p<0.05).

First, the amount of functional connectivity of the brain functional network and its relative change between phase T1–T3 in the driving state and phase T0 in the awake state from all subjects in different rhythms were studied statistically with a threshold *T* of 0.74, as is shown in Figure 6. The amount of functional connectivity we were interested in were the ones in the whole brain (Total), in the left hemisphere (LL), between the left and right brain regions (LR), and in the right hemisphere (RR). It is found that for delta, theta and alpha rhythms, the amount of functional connectivity and the relative change of Total, LR and RR were gradually increasing with the increase of simulated driving time, and reach the highest in phase T3. Particularly, the amount of functional connectivity of LR was relatively smaller than LL, LR and RR, which was usually smaller than 50 under the threshold of 0.74 at phase T0. As the driving fatigue deepened, a significant change two or three times the relative change of the functional connectivity occurred.

Furthermore, one-way ANOVA followed by Dunnett’s test (*p* < 0.05) was performed in phase T1, T2 and T3 compared to phase T0. The relative change in the functional connectivity increased significantly in LR at phase T3 for delta, theta and alpha rhythms, and in Total and RR at phase T3 for the delta rhythm. There was no significant change in the beta rhythm. The above analysis shows that the functional connectivity in LR was sensitive for analyzing driving fatigue.

### 4.3. Shortest Path Length Analysis

**Figure 7:**
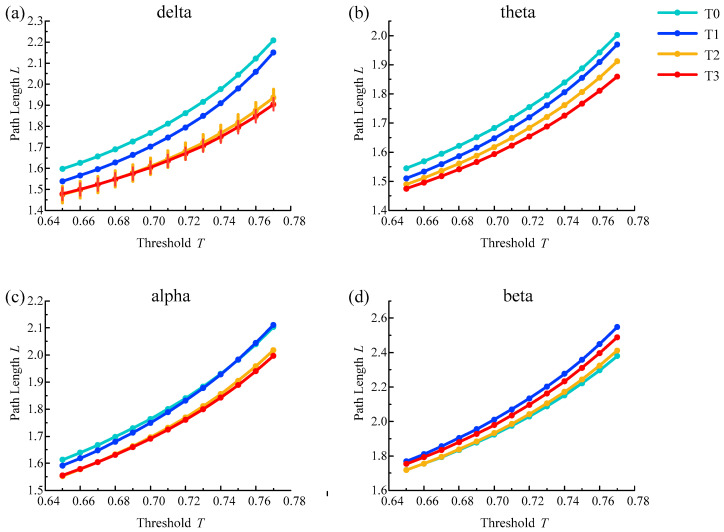
Results of the average shortest path length for (**a**) delta, (**b**) theta, (**c**) alpha and (**d**) beta rhythms. Vertical lines represent the average shortest path length with significant differences compared to phase T0 (p<0.05).

The average shortest path length results of the brain functional network from all subjects in different rhythms are shown in Figure 7. With the increase in simulated driving time, the average path length for delta, theta and alpha rhythms showed a trend of shortening except for the irregular change of the beta rhythm and reached the shortest value in phase T3 for delta, theta and alpha rhythms. In addition, the above changes were consistent under a series of selected thresholds.

Furthermore, one-way ANOVA followed by Dunnett’s test (*p* < 0.05) was performed in phase T0 of the awake state and phases T1–T3 in the driving state based on the selected thresholds. For the delta rhythm, there were statistical differences at all selected thresholds in phase T2 and T3. For other rhythms, there were no statistical differences in the selected thresholds for any of the phases. The above analysis shows that the average shortest path length is suitable for analyzing driving fatigue, especially for the delta rhythm.

### 4.4. Average Clustering Coefficient Analysis

**Figure 8:**
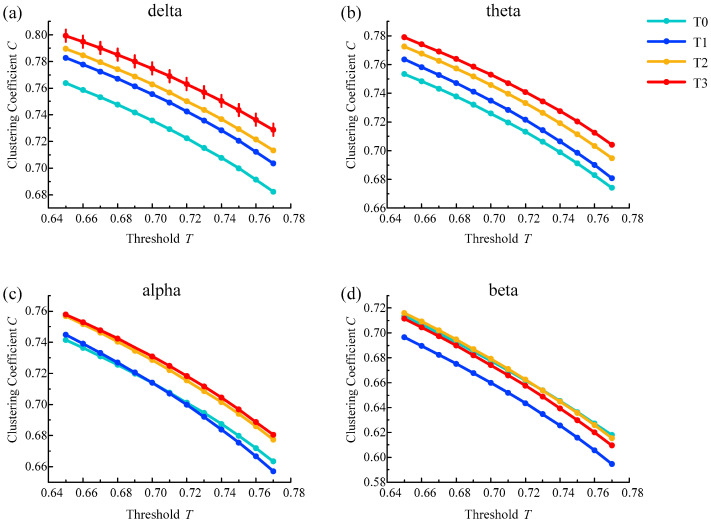
Results of the average clustering coefficient for (**a**) delta, (**b**) theta, (**c**) alpha and (**d**) beta rhythms. Vertical lines represent the average clustering coefficient with significant differences compared to phase T0 (p<0.05).

In the brain functional network, the average clustering coefficient results from all subjects in different rhythms are shown in Figure 8. The average clustering coefficient for delta, theta and alpha rhythms increased gradually with the increase in simulated driving time, except for the irregularity of the beta rhythm. The average clustering coefficient was highest in phase T3 for the delta, alpha and theta rhythms. In addition, the above changes were consistent under a series of selected thresholds.

In addition, one-way ANOVA followed by Dunnett’s test (*p* < 0.05) was performed in phase T0 of the awake state and phases T1–T3 of the driving state based on the selected thresholds. Among them, for the delta rhythm, there were statistical differences at all selected thresholds in phase T3. For other rhythms, there were no statistical differences in the selected thresholds for any of the phases. The above analysis results show that the average clustering coefficient feature was good for analyzing brain fatigue, especially for the delta rhythm.

### 4.5. Degree Centrality Analysis

**Figure 9:**
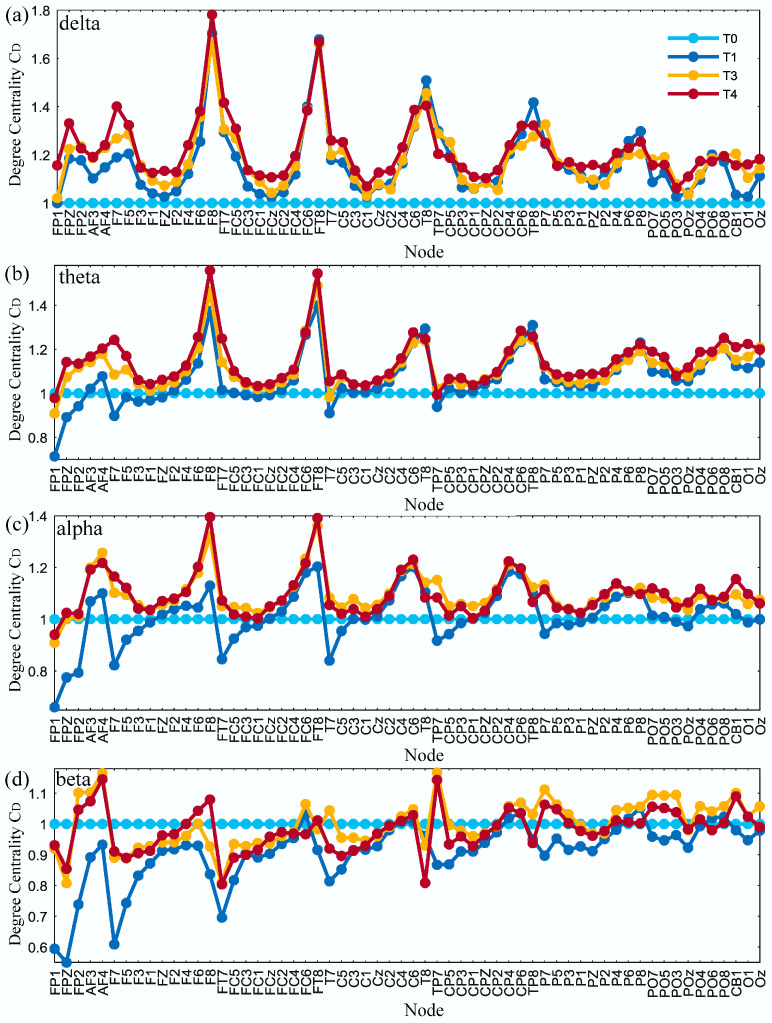
Results of the relative degree centrality for (**a**) delta, (**b**) theta, (**c**) alpha and (**d**) beta rhythms in phases T1–T3 compared to phase T0.

The average degree centrality of the brain network was obtained under the threshold of 0.74 for all subjects in phases T0–T3 as shown in Figure 9. It can be seen that, with the increase in simulated driving time, the degree centrality of partial nodes tended to increase for delta, theta and alpha rhythms. There was no obvious pattern of change for the beta rhythm.

**Figure 10:**
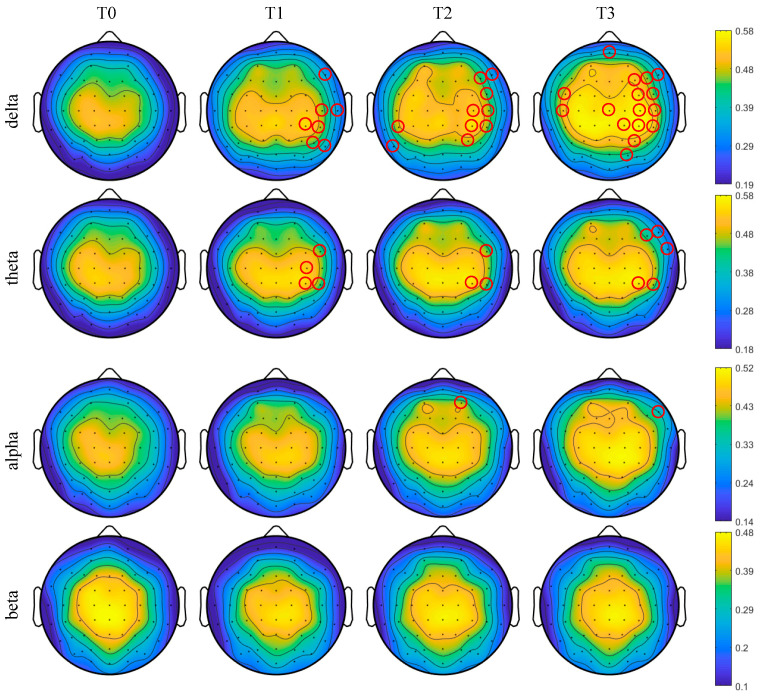
The brain topographic maps of the degree centrality for all subjects in different rhythms. Red circles represent the degree centrality with significant differences compared to phase T0 (p<0.05).

Furthermore, the brain topographic maps of the average degree centrality were obtained at the threshold 0.74 for all subjects in phases T0–T3 as shown in Figure 10. One-way ANOVA followed by Dunnett’s test (*p* < 0.05) was performed in phase T0 of the awake state and phases T1–T3 of the driving state on the selected thresholds. The red circles marked in Figure 10 are the nodes with significant differences. With the increase in simulated driving time, the number of nodes with significant differences gradually increased for delta and theta rhythms and reached the maximum in phase T3. Especially for the delta rhythm, the degree centrality significantly increased for partial channels in phase T3 in the right hemisphere, which were the frontal region, frontal-central region, central region, central-parietal region, parietal region and parietal-occipital region. For the theta rhythm, the degree centrality was significantly increased for individual channels in phases T1–T2 in the right hemisphere, which were the frontal-central region, central region and central-parietal region and in phase T3 in the right hemisphere, which were the frontal region, frontal-central region and central-parietal region. For the alpha rhythm, the degree centrality was significantly increased for AF4 channel in phase T2 in the right hemisphere, which was the pre-frontal region, and for F8 channel in phase T3 in the right hemisphere, which was frontal region. For the beta rhythm, the degree centrality shows no significant change in phases T1–T3.

## 5. Discussion

In this paper, we used the complex network theory to study the changes of EEG signals for different rhythms in different periods of simulated driving. The functional connectivity between the nodes in the brain functional network was established by calculating the Pearson correlation coefficient between two nodes for each subject. A series of thresholds were selected, and then the corresponding brain functional network was constructed. Finally, the changes of complex network characteristics such as the amount of functional connectivity, shortest path length, clustering coefficient and degree centrality in the constructed brain functional network during driving fatigue were analyzed.

The results show that there are fewer connecting edges between the channels in phase T0 at the beginning of the simulated driving experiment. At phase T0, the subjects perform their respective nerve activities in each brain region, which has low synchronization. With the increase in simulated driving time, the synchronization increases and the fatigue degree deepens gradually. The research has shown that mental fatigue occurs during a long-term cognitive task, when the brain must activate important nerve circuits to maintain body performance and prevent attention loss [34]. In the state of mental fatigue, the brain must activate more functional connectivity in order to maintain basic cognitive functions and physiological activities [35]. This is consistent with the conclusions of some studies on the brain functional connectivity in the process of driving mental fatigue [10,11,12,13]. Interestingly, this study also finds that the relative change of functional connectivity in LR brain is more significant at phase T3, taking the delta rhythm, for example, as shown in Figure 6. If the brain functional network corresponding to phase T0 is regarded as the benchmark network, the functional connectivity of the brain functional network in the process of mental fatigue is significantly enhanced in phase T3. This indicates that the subjects have experienced mental fatigue after a long period of simulated driving, and the nerve activity in each brain region is inhibited, resulting in a synchronous increase in the neural activity of the brain region compared with phase T0, while the level of attention and awakening of the brain are declining [36].

In addition, there may be a decrease in alertness when people experience brain fatigue, and the characteristic parameters of the brain network, such as path length, clustering coefficient and degree, will change regularly compared with the awake state [37]. The results of brain network analysis show that as the degree of fatigue deepens, the average clustering coefficient for the delta rhythm increases, and the average path length becomes shorter. At the same time, the degree centrality of each node has an upward trend. The coherence is significantly increased in the frontal, central, parietal and the intermediate connections regions of the brain. The average path length and clustering coefficient represent the ability of information processing and transmission in the brain from the global and local perspectives, respectively [38]. The decrease of average path length means that the overall information processing ability is improved, which is consistent with some recent research reports. Kar et al. [14] showed a decreasing trend in the characteristic path length for delta, theta, alpha and beta bands. Chua et al. [15] showed that the synchronous network had decreased path length with the accumulation of mental fatigue for the alpha frequency band. Wang et al. [10] showed a significant reduction of the global efficiency characteristic in the 36–44 Hz band, which is a parameter corresponding to the average path length; Kong et al. [16] adopted the global efficiency and path length and found that both decreased significantly in the delta and theta bands. Although the increasing and decreasing rules of the path length characteristic parameter are the same, the corresponding rhythms are different, and some even contain the beta rhythm, which is related to the state of excitement. Nevertheless, there are also some contrasting reports. Zhao et al. [13] observed a significant increase in the character path length for all EEG bands, which is the same as the result of Dimitrakopoulos et al. [17] for the theta band in a driving task and Sun et al. [18] for the alpha1 band in weighted networks. The increase in the clustering coefficient means that the connectivity between adjacent nodes is enhanced and the ability of local information processing is improved, which is consistent with many studies on fatigued driving. Kar et al. [14] showed an increasing trend in the clustering coefficient for delta, theta, alpha and beta bands, which is the same as the result of Chua et al. [15] for the alpha frequency band, Zhao et al. [13] for delta, alpha and beta bands, Wang et al. [10] in the 36–44 Hz band and Dimitrakopoulos et al. [17] for the theta band during the driving task.

During the simulated driving task, the subjects gradually feel tired, but when the vehicle deviates, the divers have to respond as quickly as possible, which indicates that the drivers have to make more effort than normal to maintain their attention [39]. Therefore, in the fatigued state, the brain must activate more or stronger functional connectivity to improve the efficiency of information processing and transmission in the cerebral cortex, so that the brain can successfully complete the simulated driving task. As more brain function regions are activated, the degree centrality of the node corresponding to the brain region increases, which coincides with the study of Kar et al. [14] in that the degree of connectivity and the corresponding brain region of the node are more important in the network. The consequence is that the brain’s energy supply is insufficient and eventually leads to fatigue. 

It is indicated that frequency division is a powerful and effective method in vigilance and driving tasks [40,41,42]. In fact, some characteristics based on the brain functional network for different frequency bands of EEG signals have been used to analyze the features of fatigue driving [10,15,43,44,45]. We hope that we can find a strong indicator for fatigue driving detection, so we have a rigorous statistical analysis of the processed data. It is found that the characteristics, such as shortest path length, clustering coefficient and degree centrality, are more sensitive, which show regular changes, especially for the delta rhythm. To our knowledge, among the limited studies on the use of brain functional network to analyze driving fatigue, most of them focus on the theta and alpha rhythms [14,15,16,17,18], because related research shows that when a person fatigues, slow wave activity increases over the entire cortex, especially for theta and alpha rhythms [46], but there is also a study indicating that fatigue mainly influences the lowest frequency band (1–3 Hz) [47]. 

In conclusion, the amount of functional connectivity, shortest path length, clustering coefficient and degree centrality were relatively sensitive to fatigue detection. Especially for the delta rhythm, there were regular changes in each characteristic. The above research can provide some effective indicators and a theoretical basis for fatigue driving monitoring. In future work, we will continue to increase the number of subjects to expand the sample size and further improve the accuracy of statistical analysis.

## Figures and Tables

**Table 1:** Brain regions vs. EEG channels.

Brain Regions	Channels
Pre-frontal	Fp1, Fpz, Fp2, AF3, AF4
Frontal	F7, F5, F3, F1, Fz, F2, F4, F6, F8
Frontal-central	FC5, FC3, FC1, FCz, FC2, FC4, FC6
Central	C5, C3, C1, Cz, C2, C4, C6
Central-parietal	CP5, CP3, CP1, CPz, CP2, CP4, CP6
Parietal	P7, P5, P3, P1, Pz, P2, P4, P6, P8
Parietal-occipital	PO7, PO5, PO3, POz, PO4, PO6, PO8
Occipital	O1, Oz, O2
Temporal	FT7, FT8, T7, T8, TP7, TP8
